# Comparing Accuracy of the Final Height Prediction Models for Elite Football Players and Developing a New Model

**DOI:** 10.14789/jmj.JMJ21-0053-OA

**Published:** 2022-06-02

**Authors:** TAKAYUKI ANDO, MASASHI NAGAO, TAKAYUKI MIYAMORI, MICHIKO DOHI, TOMOHIKO TATEISHI, HIROSHI IKEDA, MASAFUMI YOSHIMURA

**Affiliations:** 1 Graduate School of Health and Sports Science, Juntendo University, Chiba, Japan; 1 Graduate School of Health and Sports Science, Juntendo University, Chiba, Japan; 2 JFA Academy Fukushima, Tokyo, Japan; 2 JFA Academy Fukushima, Tokyo, Japan; 3 Innovative Medical Technology Research & Development Center, Juntendo University, Tokyo, Japan; 3 Innovative Medical Technology Research & Development Center, Juntendo University, Tokyo, Japan; 4 Department of Orthopaedic Surgery, Juntendo University Faculty of Medicine, Tokyo, Japan; 4 Department of Orthopaedic Surgery, Juntendo University Faculty of Medicine, Tokyo, Japan; 5 Faculty of Health Science, Juntendo University, Tokyo, Japan; 5 Faculty of Health Science, Juntendo University, Tokyo, Japan; 6 Japan Institute of Sports Sciences, Tokyo, Japan; 6 Japan Institute of Sports Sciences, Tokyo, Japan; 7 Medical Committee, Japan Football Association, Tokyo, Japan; 7 Medical Committee, Japan Football Association, Tokyo, Japan

**Keywords:** height prediction, bone age, tanner whitehouse 2, football

## Abstract

**Objective:**

This study aimed to assess the accuracy of previously developed height prediction models in male Japanese football players and create new height prediction models.

**Materials:**

The participants were elite academy male football players. We collected current height, parent's height, calendar age and bone age in 6^th^ grade of primary school and obtained actual final height at 20 to 28 years old.

**Methods:**

We compared the accuracy of two conventional models for predicting final height. These used current height, calendar age and either bone age (Model 1) or parental height (Model 2). We then developed a new model to optimize the coefficients of Model 1 (Model 3). The final model added parental height to Model 3 and optimized the coefficients (Model 4).

**Results:**

Prediction accuracy was higher for Model 2 (R = 0.52, P < 0.001) than Model 1 (p = 0.33, P < 0.001). The equation of Model 3 was final height = 0.63229313×actual measured height-8.2541327×calendar age-2.3009853×bone age (TW2)+206.627184. The R-square was 0.49 (P < 0.0001). The equation of Model 4 was final height = 0.32156081×actual measured height − 4.6652063×calendar age+0.41903909×father's height+0.34952508×mother's height-0.740469×bone age(TW2)+62.1007751. The R-square was 0.61 (P < 0.0001).

**Conclusions:**

In the two previous conventional models, a formula using parental height had better predictive accuracy. We developed a new height prediction model using current height, calendar age, father's and mother's height and bone age.

## Introduction

In some competitive sports, efforts have been made to predict future height. There have been various reports of positive correlations between height and athletic performance^1-3)^. Assessing athletic potential based on skeletal development, such as predicting future height, is therefore considered an important factor in estimating athletic talent.

Current methods of predicting final height from a single time point include models estimated with bone age and parents' height. Bone age, which measures biological bone maturity, is estimated in three ways: the numerical method^4)^, the qualitative method^5)^, and the measurement method^6)^. The numerical method counts the number of ossification centers on the hand. The qualitative method compares the shape of the ossification centers on hands to a standard chart, and the measurement method measures the area and aspect ratio of the ossification centers on the hand. For the Japanese population, Murata et al. standardized the Tanner Whitehouse 2 (TW2) method, a qualitative method^7)^.

The Growth Potential method^8)^ and the Bayley- Pinneau method^9)^ have been used to predict final height using bone age, and a prediction formula for Japanese children was developed by Matsuoka et al. in 1994.^10, 11)^. As an alternative, Ogata et al. developed the target height method, which calculates the predicted height of Japanese children based on their parents' height rather than using bone age^12)^.

In clinical practice, bone age is usually used for differential diagnosis or follow-up of growth disorders in children. Its use to predict the future height of athletes might therefore be challenging, because it requires radiography, and not many athletes have access to this. Estimates based on parental height are therefore often used. Previous studies have suggested several models to predict future height, but it is not known which formula is accurate and whether the model fits athletes. This study therefore aimed to compare the accuracy of the prediction formulas, and to develop a new prediction formula for athletes.

## Materials and Methods

This study was approved by the ethical committee of Juntendo University (No.2021-12) and was carried out from June to October 2021. Consent forms were sent to potential participants and we enrolled any who agreed to participate and provided written consent. The participants were elite academy football players who belonged to a football club linked to the Japan Football Association. They were all dormitory lives and had a similar lifestyle of eating, sleeping and practicing for 6 years.

The participants had all belonged to the football club for six years from the age of 12 to 18 (from 2006 to 2019). We collected data in October of their 6th grade of primary school (11 to 12 years old) with agreement of their parents (the fiscal year starts in April and ends in March in Japan) and then at the age of 20 to 28. In 6th grade of primary school, we obtained participants' height, weight, parents' height, and hand x-rays. X-rays were taken at a nearby clinic. At the age of 20-28, we obtained participants' current height, weight and age. Height measured after 20 years old was defined as final height and was self-reported by participants to the investigator. We excluded those who were less than 20 years old in 2021.

### Estimated bone age

We used the TW2 method^5)^ to calculate bone age. This has three types of evaluation methods: RUS (radius, ulna and short bone), carpal and 20Bone. The RUS method evaluates 13 nuclei; the carpal method evaluates seven nuclei (carpal bone); and the 20bone method evaluates 20 nuclei (both RUS and carpal). We used the RUS method, which was reported to have a high correlation with final height^11)^, to calculate the bone age.

### Models predicting future height

First, we compared two conventional models. The first model (Model 1) was reported by Matsuoka et al. in 1994^11)^. The formula was developed using actual measured height, calendar age and bone age, and the equation was Y = a × actual measured height + b × calendar age + c × bone age + d. In the model, a, b, c and d change depending on the calendar age. In this study, for those with a calendar age of 11.5 to 12 years, we used Y = 0.639 × actual measured height − 9.221 × calendar age − 3.567 × bone age + 230.39. We used Y = 0.557 × actual measured height + 7.809 × calendar age − 3.413 × bone age + 36.161 for those with a calendar age of 12 to 12.5 years, and Y = 0.288 × actual measured height + 6.475 × calendar age − 1.825 × bone age + 36.161 for those aged 12.5 to 13 years. Actual measured heights at 6^th^ grade of primary school were measured on the day of the hand X-ray. We measured the left hand^12)^ unless it had previously been broken. The second model (Model 2) was the target height method developed by Ogata et al. in 1990^13)^. The formula uses parents' height and the equation was (father's height + mother's height + 13)/2.

After assessing Models 1 and 2, we created new height prediction models, Models 3 and 4. We conducted a multiple regression analysis using the same variables as Model 1 and developed a new equation by changing coefficients, to give Model 3. We then developed another prediction model with different variables (Model 4). To develop this final model, we used regression analyses, added or removed variables and calculated the accuracy. The final Model 4 included actual measured height, calendar age, father's height, mother's height and bone age.

### Statistical analysis

Multiple regression analysis used the statistical software JMP^®^ pro version 16. The significance level of the test was set at 5%.

## Results

### Participants

Of the 128 male players who belonged to the football club from 2006 to 2019, 53 men agreed to participate and were included in this study. The demographic characteristics of the participants are shown in Table 1. The mean (SD) bone age calculated by the TW2 method was 12.2 (1.2). The mean difference (SD) between bone age and calendar age was 0.89 (0.75) years, and the mean difference between final height and measured height in 6^th^ grade of primary school was 22.9 (8.2).

**Table 1:** Characteristic of the study participants (N = 53)

	Mean (SD)
**Data in 6^th^ grade of primary school**	
Calendar Age (years)	12.2 (0.3)
Bone age (years)	12.2 (1.2)
Height (cm)	149.2 (9.7)
Predicted height of Model 1 (cm)	173.2 (4.5)
**Data in adults (20-28years old)**	
Age (years)	23.9 (2.8)
Actual final height (cm)	172.1 (6.8)
Father’s height (cm)	173.5 (5.4)
Mother’s height (cm)	158.3 (5.3)

### Difference between predicted height and final height

**Figure 1:**
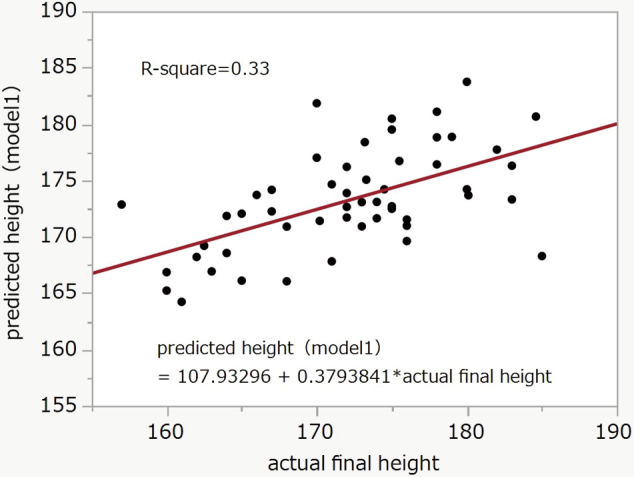
Correlation between final and predicted height (Model 1) R-square = 0.33, P < 0.0001

**Figure 2:**
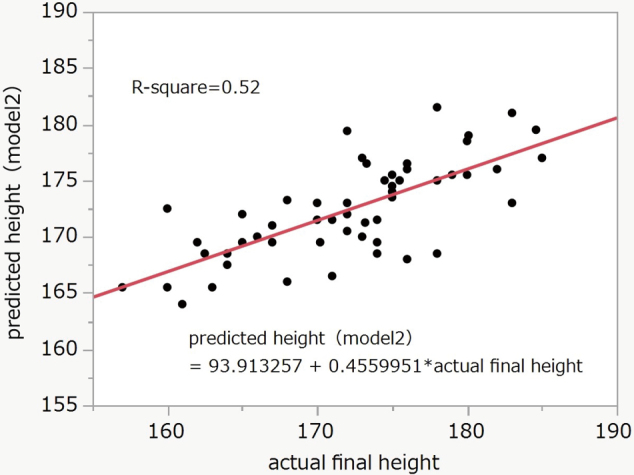
Correlation between final and predicted height (Model 2) R-square = 0.52, P < 0.0001

We assessed the accuracy of the conventional models by correlation analysis. For Model 1, the mean (SD) of the predicted heights was 173.2 (4.5) and the actual final height was 172.1 (6.8). The R-square was 0.33 (P < 0.0001) (Figure 1). For Model 2, the mean (SD) of the predicted heights was 172.4 (4.3) and the actual final height was 172.1 (6.8). The R-square was 0.52 (P < 0.0001) (Figure 2).

### New prediction models

**Figure 3:**
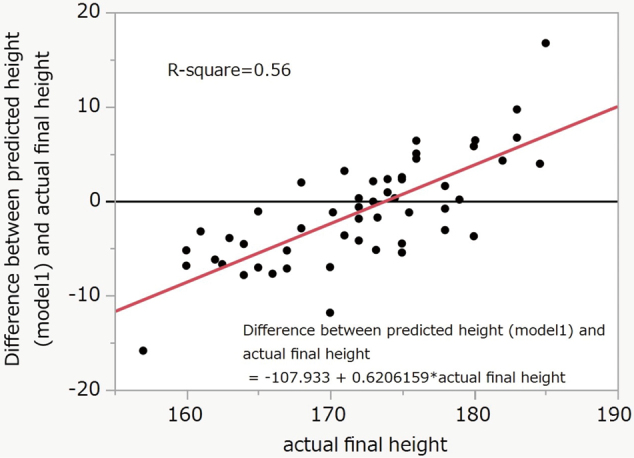
Difference between final and predicted height (Model 1) R-square = 0.56, P < 0.0001

**Figure 4:**
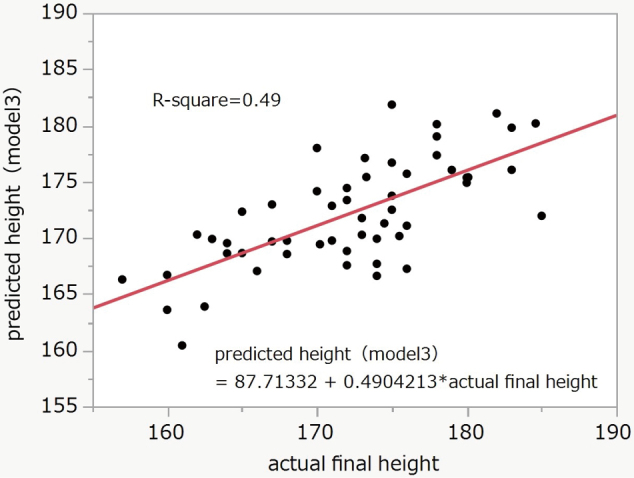
Correlation between final and predicted height (Model 3) R-square = 0.49, P < 0.0001

Model 2 was more accurate than Model 1. Model 1 was accurate at a final height of around 170–175cm. However, the difference was more prominent below 170cm or above 175cm. The difference between the predicted height and actual final height was linearly distributed, and the R-square was 0.56 (P < 0.0001) (Figure 3). The equation of Model 3 was final height = 0.63229313 × actual measured height − 8.2541327 × calendar age − 2.3009853 × bone age (TW2) + 206.627184. The R-square was 0.49 (P < 0.0001) (Figure 4).

**Figure 5:**
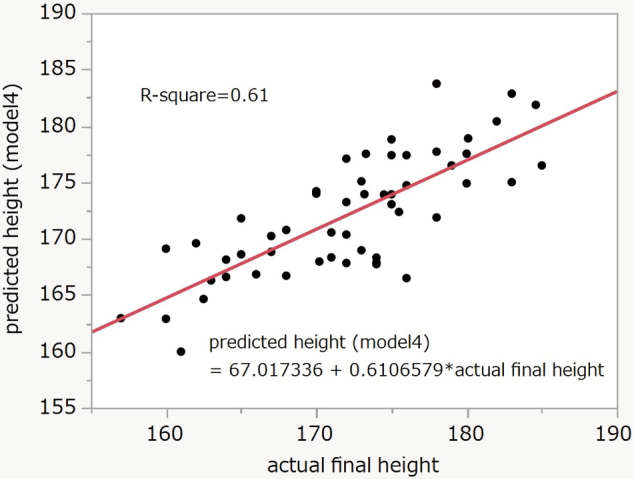
Correlation between final and predicted height (Model 4) R-square = 0.61, P < 0.0001

Finally, we added the height of the parents as a parameter and as a result, it showed higher accuracy than model 2 (model 4). The equation was final height = 0.32156081 × actual measured height − 4.6652063 × calendar age + 0.41903909 × father's height + 0.34952508 × mother's height − 0.740469 × bone age (TW2) + 62.1007751. The R-square was 0.61 (P < 0.0001) (Figure 5).

## Discussion

In this study, we assessed the accuracy of the two conventional models of final height prediction for Japanese young people, in a group of football players. One model uses bone age, calendar age, and actual measured height as variables and the other uses the parents' height. We demonstrated that the model using parents' height was more accurate (R-square was 0.52 compared with the bone age model developed by Matsuoka et al.). In the bone age model developed by Matsuoka et al., accuracy was good for a final height of between 170 to 175 cm, but less accurate outside this range. The discrepancy was distributed linearly, and we therefore optimized the coefficients with the same four variables. The R-square became 0.49, but the accuracy was still lower than using the parents' heights (R-square = 0.52). In a new model with bone age added as well as parental height, the R-square became 0.61. As a result, a new predictive model by changing the coefficients of the bone age model developed by Matsuoka et al. and adding the height of the parents as variables demonstrated better accuracy. According to our data, the prediction model that did not use bone age was less accurate than our newly developed model. However, we think that the parental height model developed by Ogata et al. has sufficient accuracy and could be used in athletes without radiation exposure.

This study aimed to develop prediction models at the 6th grade of primary school. Although several models for predicting final height using bone age have been reported^14-16)^, the accuracy varies. Models with low prediction accuracy uses bone age at a single point, while those with high accuracy use changes in height and bone age over a period. As individuals' growth speed differs^17, 18)^, taking into account the growth process may improve accuracy. However, previous reports showed that considering growth curves did not have good accuracy. Therefore, how we consider the growth process is needed to be investigated. We should note that data of body weight was obtained in the study; however, it was not helpful to predict future height. Therefore we did not use the variable.

This study had several limitations. First, we enrolled 53 participants to compare the accuracy of traditional models and develop new models, but we did not calculate a required sample size. However, all the data in the study have a low p-value of less than .05, and type 2 errors therefore should not be a problem. Second, this new model may not fit non- elite athletes. In this study, the mean final height was 172.1cm, which is slightly taller than the mean of all Japanese men^19)^. The mean parental heights in this study were also greater than the population mean. The height of athletes may have been taken into account, as well as their playing skills, in deciding on elite status. This may have introduced selection bias, and may make this model suitable only for a limited group of people. Third, we did not assess whether this model fits future athletes because we used all data to develop a new model. Another data set is needed to validate these models.

In conclusion, we showed that a conventional model of future height prediction using parents' height was more accurate than those using bone age, and we developed a new and more accurate height prediction model using actual measured height, calendar age, father's height, mother's height and bone age.

## Funding

The authors received no financial support for the research.

## Authors' contributions

Conception and design of the study: TA, MN, TM, MY

Research data collection: TA, MD, HI, TT

Analysis and interpretation of data: TA, MN

Writing the paper: TA, MN

Approval of final manuscript: All authors

## Conflicts of interest statement

The authors have no conflict of interest to disclose.
